# Involvement of α7nAChR in the hepatic-protective effect of remifentanil preconditioning in ischemia/reperfusion rats

**DOI:** 10.1186/s41065-025-00601-6

**Published:** 2025-12-29

**Authors:** Chaoxiong Zhou, Yuan Ma, Da Qiu, Qianjin He, Qinshu Xiao, Yaohua Wu, Quanshui Hao, Huaping Wang

**Affiliations:** 1 Department of Hepatobiliary Surgery, Huanggang Central Hospital, Huanggang, 438000 Hubei Province China; 2 School of Medicine, Wuhan University of Science and Technology, No. 10, West Huangjiahu Road, Hongshan District, Wuhan, 430060 Hubei Province China; 3 Department of Anesthesiology, Huanggang Central Hospital, No. 16, Qi ’an Avenue, Huangzhou District, Huanggang, 438000 Hubei Province China

**Keywords:** Hepatic ischemia-reperfusion injury, Remifentanil, α7 nicotinic acetylcholine receptor, Nuclear factor-kappa b, Inflammation

## Abstract

Hepatic ischemia-reperfusion (I/R) injury is an unavoidable process in liver surgeries like transplantation and hepatectomy, and it greatly impairs postoperative liver function. Recent studies have shown that anesthetic agents, such as remifentanil, offer liver protection. However, the exact mechanisms of remifentanil’s protective effects remain unclear. In this study, a rat hepatic I/R injury model and a hepatocyte BRL-3 A hypoxia/reoxygenation (H/R) model were successfully established, and remifentanil preconditioning (RPC) was administered. Liver function enzyme activities were measured using biochemical assays, and the extent of liver damage was assessed via HE staining as well as TUNEL staining. The survival rate and apoptosis rate of BRL-3 A cells were determined by CCK-8 assay and flow cytometry, respectively. Levels of proinflammatory cytokines were quantified using ELISA. qRT-PCR and Western blotting analysis were employed to evaluate the expression of α7 nicotinic acetylcholine receptor (α7nAChR) and the phosphorylation of NF-κB. Loss-of- function experiments of α7nAChR were conducted to further elucidate the underlying molecular mechanism by which remifentanil protects the liver against I/R injury. The findings demonstrated that RPC markedly mitigated liver dysfunction, decreased hepatic cell necrosis and apoptosis, and suppressed the inflammation in rats subjected to I/R. Furthermore, RPC provided protection for BRL-3 A cells against H/R-induced injury and inflammation. Notably, RPC upregulated α7nAChR expression in I/R liver tissue and H/R-exposed BRL-3 A cells while concurrently inhibiting NF-κB phosphorylation. However, the protective effects of RPC on hepatic I/R injury and H/R-induced BRL-3 A cells injury were abrogated by the administration of an α7nAChR antagonist or α7nAChR knockdown. Taken together, these data reveal a novel mechanism of remifentanil’s hepatoprotective effect, which is that remifentanil alleviates hepatic I/R injury by upregulating α7nACh to inhibit inflammation mediated by NF-κB activation.

**Supplementary Information:**

The online version contains supplementary material available at 10.1186/s41065-025-00601-6.

## Introduction

Hepatic ischemia-reperfusion (I/R) injury represents a significant complication following liver transplantation, trauma, and hepatic tumor resection, which impairs postoperative liver function recovery and may lead to liver dysfunction [1]. The underlying pathogenesis is multifaceted, encompassing inflammatory responses, oxidative stress, mitochondrial dysfunction, and additional contributing factors [2, 3]. Nevertheless, conventional anti-inflammatory and antioxidant drugs are limited by poor solubility, short circulation half-life, and severe side effects, thereby restricting their efficacy in managing hepatic I/R injury [4].

The alpha 7 nicotinic acetylcholine receptor (α7nAChR) is a ligand-gated ion channel and represents one of the most distinctive members of the cys-loop superfamily. It is composed of five identical α7 subunits arranged in a homologous pentameric structure, exhibiting high permeability to calcium ions [5, 6]. The α7nAChR is extensively distributed on the membranes of neurons, endothelial cells, immune cells, and other cell types. As a critical receptor in the cholinergic anti-inflammatory pathway, it plays a pivotal role in mitigating tissue damage by reducing inflammatory responses, oxidative stress, and apoptosis [7]. Upon activation of the cholinergic anti-inflammatory pathway, its primary downstream effect involves inhibiting the activation of the nuclear factor-kappa B (NF-κB) signaling pathway [8]. Therefore, the activation of α7nAChR can effectively suppress the nuclear translocation and activity of NF-κB, thereby decreasing the production and release of pro-inflammatory cytokines and mitigating both local and systemic inflammatory responses [9, 10]. Additionally, the activation of α7nAChR can alleviate oxidative stress in tissues via multiple mechanisms, such as inhibiting pro-inflammatory factors, enhancing the activity of endogenous antioxidant enzymes, and activating the Nrf2 signaling pathway [11–13]. Given that oxidative stress and inflammatory factors are critical drivers of apoptosis, the activation of α7nAChR can also indirectly reduce apoptotic processes [14, 15]. The activation of α7nAChR can attenuate the systemic inflammatory response in rats with myocardial I/R injury, thereby protecting the injured myocardium [16]. Furthermore, upregulation of α7nAChR expression has been shown to alleviate cognitive dysfunction in rats with cerebral ischemia by inducing autophagy and inhibiting oxidative stress [17]. Of particular significance, α7nAChR agonists mitigate hepatic I/R injury through the inhibition of the inflammatory response mediated by the activation of the NF-κB signaling pathway following hepatic I/R injury [18]. Consequently, enhancing α7nAChR activation represents a promising therapeutic strategy for the treatment of organ I/R injury.

Currently, numerous studies have demonstrated that certain anesthetic agents exhibit protective effects against organ I/R injury, including remimazolam [19], sevoflurane [20], etomidate [21], and remifentanil [22]. Remifentanil is a µ-opioid receptor agonist and a synthetic derivative of fentanyl. Owing to its distinctive pharmacokinetic characteristics—including rapid onset of action, a short half-life, and independence from hepatic and renal metabolism—it is extensively utilized in clinical anesthesia and analgesia. Furthermore, remifentanil has been shown to exert protective effects on I/R injury in various organs, including the brain [23], myocardium [24], and liver [25], through mechanisms involving significant anti-oxidative, anti-inflammatory, and anti-apoptotic properties. However, it remains unclear whether remifentanil alleviates NF-κB-mediated inflammatory responses via regulation of the α7nAChR, thereby mitigating hepatic I/R injury. Consequently, in this study, both in vivo and in vitro rat liver I/R models were established to evaluate the protective effects of remifentanil on hepatic I/R injury. Additionally, an α7nAChR antagonist was employed to further elucidate the molecular mechanisms underlying remifentanil’s role in reducing hepatic I/R injury. This investigation aims to provide a more robust theoretical foundation for the clinical application of remifentanil in protecting against liver I/R injury.

## Materials and methods

### Animals

Male Sprague-Dawley rats, aged 8 to 9 weeks and weighing 250 to 280 g, were supplied by the Laboratory Animal Center of Hubei Province. The rats were housed in a barrier-controlled environment with ad libitum access to food and water. All animal experiments conducted in this study adhered to the fundamental ethical guidelines for animal welfare and were approved by the Ethics Committee of Huanggang Central Hospital (approval number: HG-KY-2024-035).

### Hepatic I/R injury in vivo

Rats were subjected to a 16-h fasting period prior to surgery and were anesthetized via intraperitoneal injection of 2% sodium pentobarbital (50 mg/kg). A midline abdominal incision was performed to expose the liver, and the corresponding vascular pedicles of the branches of the portal vein and hepatic artery were occluded using non-invasive vascular clips. The middle lobe and left lobe of the liver underwent ischemia for 45 min, during which a color change from red to off-white was observed, confirming successful ischemia. Subsequently, the vascular clips were removed, and blood perfusion was restored for 120 min [22]. Blood samples were collected via orbital sinus puncture, followed by euthanasia of the rats, after which liver tissues were promptly harvested. In the sham-operated group, the abdominal cavity was immediately closed following liver exposure without performing any ischemia-reperfusion procedures. Remifentanil hydrochloride (Nhwa, Jiangsu, China) was administered via intravenous infusion at doses of 0.4, 2, and 10 µg/kg/min for 15 min, starting 25 min before hepatic I/R surgery, followed by a 10-min withdrawal period. The α7nAChR antagonist Methyllycaconitine (MLA, 5 mg/kg; MedChemExpress, Monmouth Junction, USA) was injected intravenously 5 min before remifentanil treatment. There doses have been shown to be effective and safe in previous studies [22, 26].

### Histopathological observation

The left lobe of the rat liver was fixed via immersion in paraformaldehyde for 24 h and subsequently processed into paraffin-embedded sections. Following deparaffinization and hydration, the sections were stained with hematoxylin and eosin (HE; Servicebio, Wuhan, China). Microscopic examination was then performed to evaluate the pathological changes in the rat liver tissues.

### TdT-mediated dUTP nick-end labeling (TUNEL) staining

Paraffin-embedded sections of rat liver tissue were prepared, deparaffinized with xylene and gradient ethanol, and subsequently treated with proteinase K for antigen retrieval. Subsequently, permeabilization was achieved by applying the membrane- breaking working solution onto the sections. The prepared TUNEL reaction solution (Servicebio, Wuhan, China) was then applied to the samples. Nuclei were counterstained with DAPI (Servicebio, Wuhan, China), and the sections were subsequently sealed. Finally, the degree of apoptosis in rat liver tissue was examined under a fluorescence microscope.

### Cell culture and infection

Rat normal hepatocytes (BRL-3 A) were obtained from Cellverse Co., Ltd. (Shanghai, China) and maintained in Minimum Essential Medium (MEM; Gibco, Carlsbad, USA) supplemented with 10% fetal bovine serum (FBS; Gibco, Carlsbad, USA) and 1% penicillin-streptomycin solution (Cellverse, Shanghai, China). Three short hairpin RNAs (shRNAs) were designed based on the reference mRNA sequence of α7nAChR (NM_012832.4) provided by the NCBI database. Following packaging, purification, and titer determination, the sh-α7nAChR lentivirus was used to infect BRL-3 A cells in the presence of Polybrene at a multiplicity of infection (MOI) of 30. The expression level of α7nAChR in the infected cells was evaluated 48 h post-infection to identify the lentivirus with the highest interference efficiency for subsequent experiments. The shRNA sequences of α7nAChR are as follows:

sh-α7nAChR-1#: 5’-CAGTGATTGTGCTGAGATATTCAAGAGATATCTCAGCAC AATCACTGTTTTT-3’;

sh-α7nAChR-2#: 5’-GCAGATATCAGCAGCTATATTCAAGAGATATAGCTGCTG ATATCTGCTTTTT-3’;

sh-α7nAChR-3#: 5’-CAGACATTCTCCTCTATAATTCAAGAGATTATAGAGGAG AATGTCTGTTTTT-3’.

### Hepatocyte hypoxia-reoxygenation (H/R) injury model in vitro

BRL-3 A cells were seeded in 6-well plates at a density of 1 × 10^5^ cells per well. Following 24 h of culture, when the cell confluence reached approximately 80%, the cells were exposed to hypoxic medium and incubated under anoxic conditions (comprising 1% O_2_, 5% CO_2_, and 94% N_2_) for 6 h. Subsequently, the cells were transferred to normal medium and cultured under normoxic conditions (5% CO_2_ and 95% air) for an additional 6 h [27].

### CCK-8 assay

BRL-3A cells were seeded into 96-well plates at a density of 3 × 10^3^ cells per well and cultured until the cells adhered to the wall. Subsequently, the cells were pretreated with remifentanil at various concentrations (0.1, 1, 10, and 100 ng/mL) for 60 min prior to H/R exposure. Upon completion of the treatment, 10 µL of CCK-8 solution (Beyotime, Shanghai, China) was added to each well and incubated for 2 h. Finally, the absorbance at 450 nm was measured using a microplate reader, and the cell viability was calculated based on the obtained data.

### Flow cytometry

The density of BRL-3 A cells was adjusted to 1 × 10^6^ cells/mL. Subsequently, 100 µL of the cell suspension was centrifuged and resuspended in 195 µL of binding buffer. 5 µL of Annexin V-FITC and 5 µL of propidium iodide (PI) staining solution (Beyotime, Shanghai, China) were added, followed by gentle mixing and incubation in the dark at room temperature for 15 min. Finally, the extent of apoptosis was quantified using flow cytometry.

### Enzyme-linked immunosorbent assay (ELISA)

Rat serum samples and BRL-3 A cell culture supernatants were collected, and the concentrations of interleukin 1β (IL-1β), IL-6, and tumor necrosis factor α (TNF-α) in both the rat serum and BRL-3 A supernatant were measured following the experimental protocols in the ELISA kit’s instructions (Elabscience, Wuhan, China).

### Biochemical assay

Rat serum and BRL-3 A cells culture medium supernatant were collected, and the levels of lactate dehydrogenase (LDH), alanine aminotransferase (ALT), and aspartate aminotransferase (AST) in the samples were quantified using the colorimetric method, following the manufacturer’s instructions provided in the kits (Nanjing Jiancheng, Nanjing, China).

### Quantitative reverse transcription polymerase chain reaction (qRT-PCR)

TRIzol reagent (Invitrogen, Carlsbad, USA) was employed to isolate total RNA from rat liver tissue and BRL-3A cells. Subsequently, the synthesis of first-strand cDNA was performed using the 1 st Strand cDNA Synthesis Kit (ELK Biotechnology, Wuhan, China) according to the manufacturer’s instructions. Real-time PCR analysis was conducted using SYBR Green PCR SuperMix (ELK Biotechnology, Wuhan, China). The cycling conditions were as follows: 95 °C for 30 s, followed by 40 cycles of 95 °C for 10 s, 58 °C for 30 s, and 72 °C for 30 s. The primer sequences used for this experiment were: α7nAChR forward primer 5’-TCTGTGCCCTTGATAGCACAAT A-3’ and reverse primer 5’-GGCATTTTGCCACCATCAGG-3’; GAPDH forward primer 5’-ACAGCAACAGGGTGGTGGAC-3’ and reverse primer 5’-TTTGAGGGT GCAGCGAACTT-3’. The relative expression level of α7nAChR in the samples was quantified using the 2^−ΔΔCt^ method.

### Western blotting analysis

RIPA lysis buffer (Thermo Scientific, Wilmington, USA) was utilized to extract total proteins from rat liver tissues and BRL-3 A cells. The protein concentration was subsequently determined. Proteins were then separated by gel electrophoresis. After transferring the proteins onto membranes and blocking, the membranes were incubated with primary antibodies in an incubation chamber at 4 °C overnight. This was followed by incubation with secondary antibodies for 50 min at room temperature. Protein bands were visualized using a chemiluminescence reagent applied to the membrane, and the grayscale values of the protein bands were analyzed using ImageJ software. The primary antibodies employed in this experiment included α7nAChR (1:1000; Santa Cruz Biotechnology, Dallas, TX, USA), NF-κB p65 (1:1000; Cell Signaling Technology, Danvers, USA), phospho-NF-κB p65 (Ser536) (1:1000; Cell Signaling Technology, Danvers, USA), and GAPDH (1:2000; Abcam, Cambridge, UK). The antibodies for NF-κB p65 and p-NF-κB p65 were used on separate membranes.

### Statistical analysis

Statistical analysis was performed using GraphPad Prism 8 software. For comparisons among multiple groups, one-way analysis of variance (ANOVA) followed by the post-hoc Tukey test was employed. A *p* < 0.05 was considered statistically significant.

## Results

### Remifentanil preconditioning (RPC) attenuated liver injury and up-regulated α7nAChR expression in rats with hepatic I/R

**Fig. 1:**
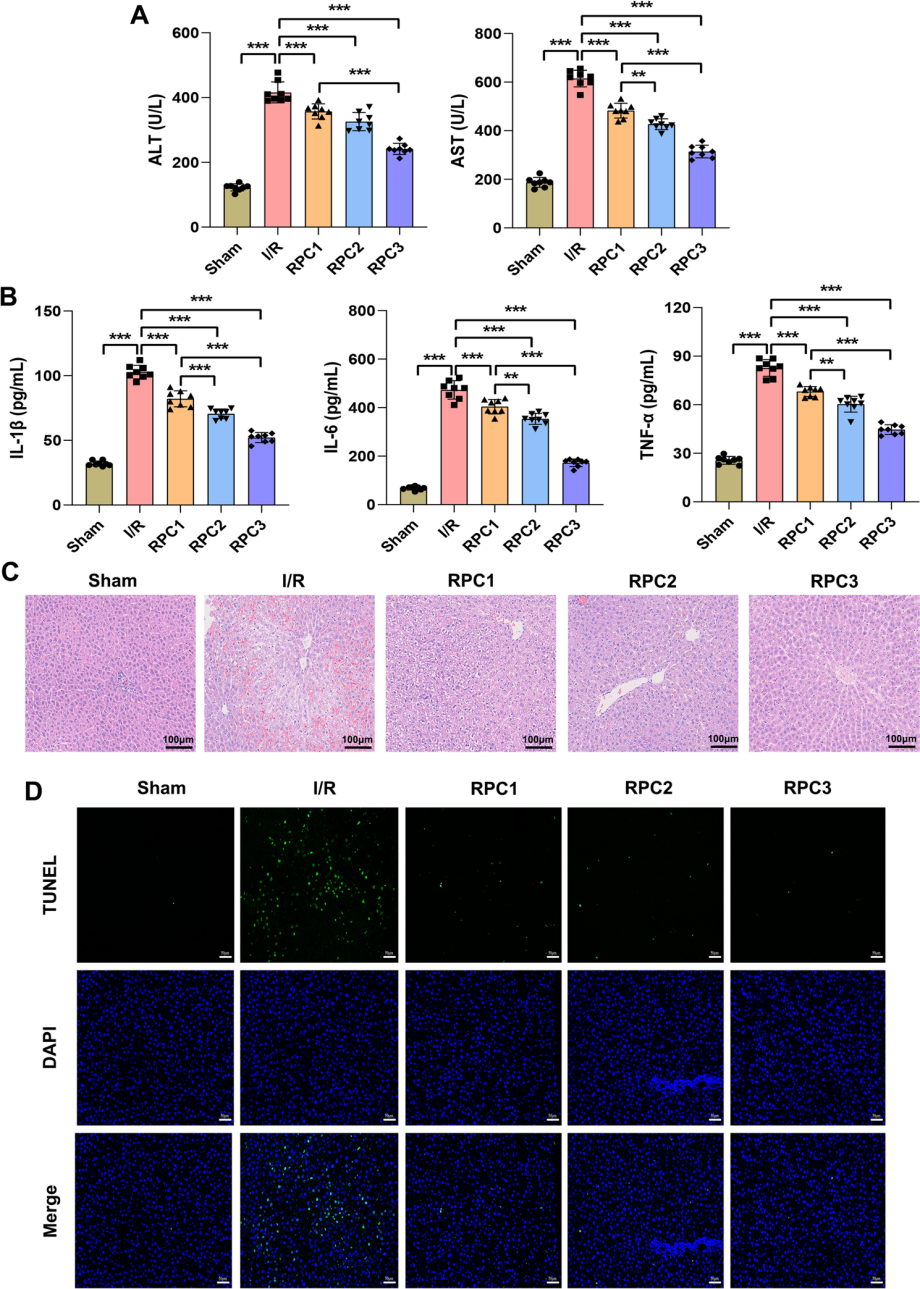
RPC reduced liver injury in I/R rats. **A** biochemical assay was used to quantify the activities of ALT and AST in serum. **B** ELISA was utilized to measure the concentrations of IL-1β, IL-6, and TNF-α in serum. **C** HE staining was performed to examine the pathological alterations in liver tissue. Scale bar = 100 μm. **D** TUNEL staining was utilized to evaluate the apoptosis of hepatocytes. Scale bar = 50 μm. RPC, remifentanil preconditioning. RPC1, RPC2 and RPC3 were defined as 0.4, 2 and 10 µg/kg/min remifentanil preconditioning, respectively. *n* = 8. ** *p* < 0.01, *** *p* < 0.001

Rats subjected to hepatic I/R injury demonstrated compromised liver function accompanied by pronounced inflammatory responses, as evidenced by markedly elevated serum ALT and AST activities (Fig. 1A) and increased concentrations of proinflammatory cytokines (IL-1β, IL-6, and TNF-α) (Fig. 1B). These effects were mitigated in rats pretreated with remifentanil prior to hepatic I/R, as reflected by reduced serum levels of ALT, AST, and proinflammatory cytokines. In addition, I/R injury significantly impaired rat liver tissue. Compared with the normal liver tissue in the Sham group, the liver tissue of rats subjected to hepatic I/R exhibited localized necrosis, abnormal hepatocyte morphology, indistinct boundaries, nuclear pyknosis (Fig. 1C), and an increased number of apoptotic cells within the tissue (Fig. 1D). RPC alleviated liver injury in I/R rats. Moreover, the improvement of liver injury was partly dose-dependent with remifentanil. In summary, RPC is capable of attenuating the inflammatory response, safeguarding liver function, and ameliorating liver injury in rats subjected to I/R.

**Fig. 2:**
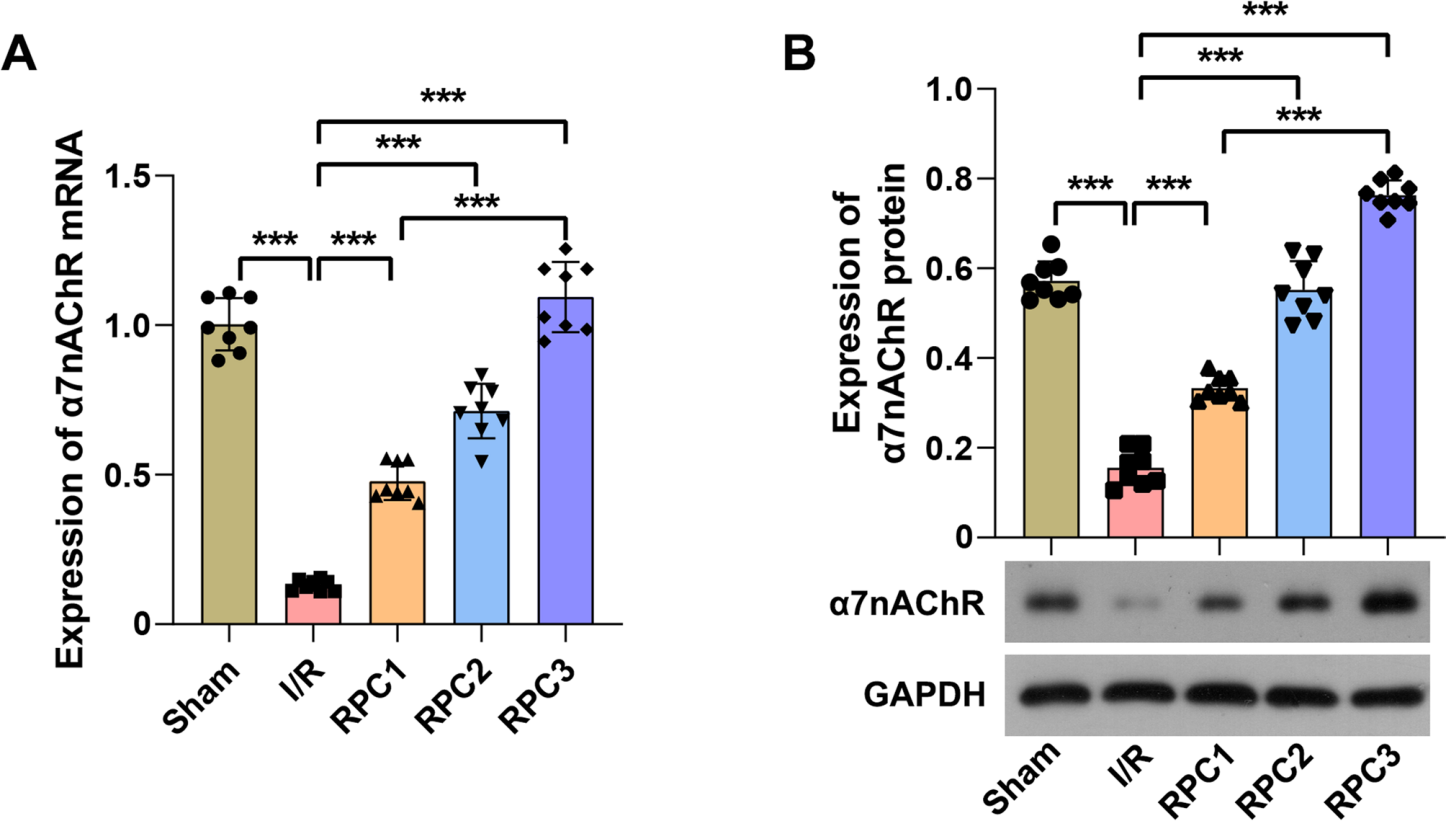
RPC promoted the expression of α7nAChR in liver tissue of rats subjected to I/R injury. **A** qRT-PCR was employed to quantify the expression level of α7nAChR mRNA in rat liver tissue. **B** Western blotting analysis was utilized to assess the protein expression of α7nAChR in rat liver tissue. RPC, remifentanil preconditioning. RPC1, RPC2 and RPC3 were defined as 0.4, 2 and 10 µg/kg/min remifentanil preconditioning, respectively. *n* = 8. *** *p* < 0.001

Subsequently, we observed that the expression of α7nAChR was significantly downregulated in the injured liver tissues of I/R rats. In contrast, following RPC, the mRNA and protein levels of α7nAChR in the liver tissues of I/R rats were markedly increased in a dose-dependent manner (Fig. 2A and B). Based on these experimental findings, we hypothesize that RPC may attenuate I/R-induced liver injury in rats by upregulating the expression of α7nAChR.

### Inhibition of α7nAChR attenuated the hepatic-protective effect of RPC in rats subjected to I/R injury

**Fig. 3:**
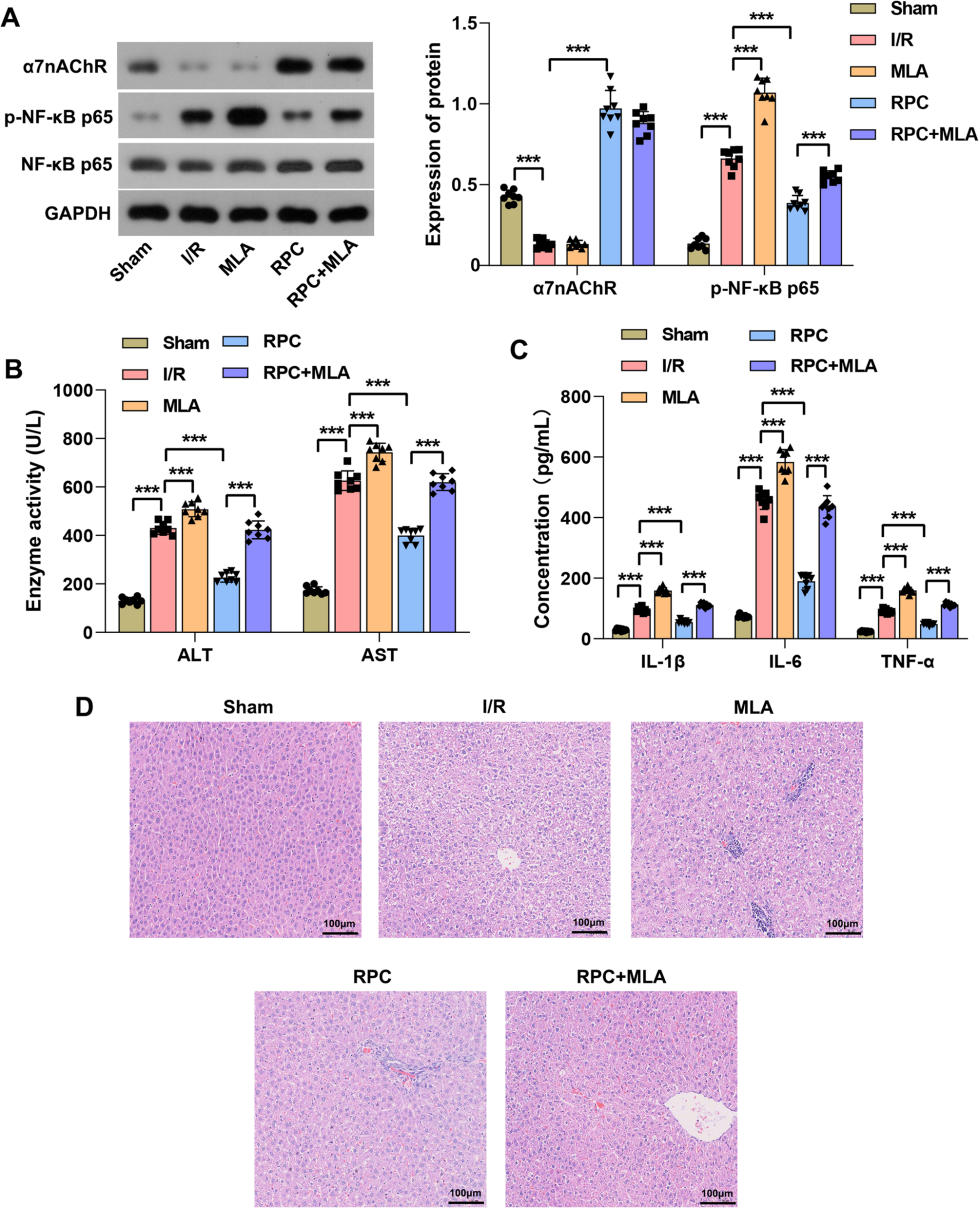
α7nAChR antagonist partially attenuated the protective effect of RPC on liver injury in rats with I/R. **A** Western blotting analysis was employed to assess the protein expression levels of α7nAChR, NF-κB p65, and p-NF-κB p65 in rat liver tissue. **B** Biochemical assay was used to quantify the activities of ALT and AST in rat serum. **C** ELISA was employed to determine the concentrations of IL-1β, IL-6, and TNF-α in rat serum. **D** HE staining was utilized to evaluate the pathological damage in liver tissue. Scale bar = 100 μm. RPC was defined as 10 µg/kg/min remifentanil preconditioning. *n* = 8. *** *p* < 0.001

To evaluate the accuracy of the aforementioned hypothesis, we employed the α7nAChR antagonist MLA to inhibit α7nAChR channel activity in liver tissue from I/R rats while administering high-dose remifentanil as a pretreatment. The results demonstrated that RPC enhanced the expression of α7nAChR protein and reduced the phosphorylation level of NF-κB p65 in I/R rat liver tissue. The NF-κB signaling pathway represents one of the key downstream pathways of α7nAChR. Treatment with MLA did not reverse the I/R-induced downregulation of α7nAChR expression but instead promoted the phosphorylation of NF-κB p65. Furthermore, MLA diminished the inhibitory effect of RPC on NF-κB p65 phosphorylation (Fig. 3A). Interestingly, the treatment with MLA resulted in a further increase in the activities of ALT and AST (Fig. 3B), as well as the serum levels of IL-1β, IL-6, and TNF-α in I/R rats (Fig. 3C). Moreover, the inhibitory effect of RPC on these indices was partially reversed by the concurrent MLA treatment. Additionally, MLA treatment exacerbated the pathological damage to liver tissue in I/R rats, increased the extent of hepatocyte necrosis, and diminished the protective effects of RPC on I/R-induced liver injury (Fig. 3D). Together, these results indicate that RPC mitigates liver injury in I/R rats, partly by modulating the α7nAChR/NF-κB p65 axis to suppress inflammatory responses.

###  Remifentanil alleviated hepatocyte injury induced by H/R through the up-regulation of α7nAChR expression in vitro

**Fig. 4:**
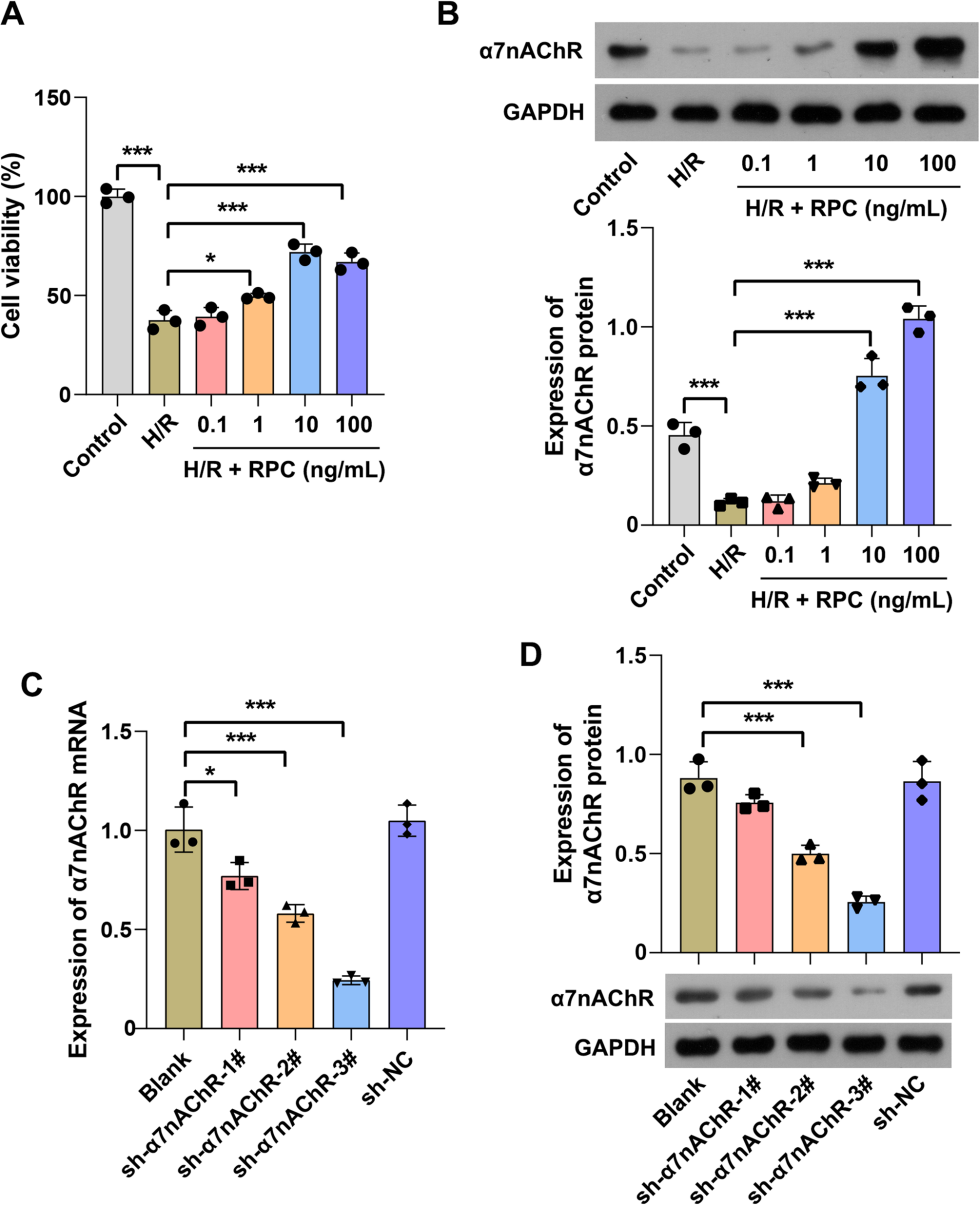
RPC up-regulated the protein expression of α7nAChR in BRL-3 A cells exposed to H/R. BRL-3 A cells were pretreated with remifentanil at various concentrations (0.1, 1, 10, and 100 ng/mL) for 60 min and subsequently subjected to hypoxic conditions for 6 h followed by reoxygenation for 6 h. **A** Cell viability was assessed using the CCK-8 assay. **B** The expression of α7nAChR protein in BRL-3 A cells was evaluated by Western blotting analysis. **C**-**D** Following infection of BRL-3 A cells with α7nAChR-interfering lentivirus, qRT-PCR and Western blotting were employed to determine the mRNA and protein expression levels of α7nAChR. RPC, remifentanil preconditioning. *n* = 3. * *p* < 0.05, *** *p* < 0.001

In the in vitro study, H/R-induced hepatocyte injury was utilized to simulate the pathological state of hepatocytes under I/R conditions. The proliferation of BRL-3 A cells was markedly suppressed by H/R stimulation. However, RPC significantly enhanced the proliferation of BRL-3 A cells exposed to H/R in a concentration- dependent manner (Fig. 4A). Furthermore, consistent with the aforementioned in vivo results, the protein expression level of α7nAChR was substantially reduced in BRL-3 A cells subjected to I/R. RPC increased this protein expression level in a concentration-dependent manner (Fig. 4B). Given that pretreatment with 100 ng/mL remifentanil yielded similar hepatocyte survival rates under H/R exposure as 10 ng/mL, we opted for a 10 ng/mL remifentanil intervention to minimize potential cytotoxic effects.

**Fig. 5:**
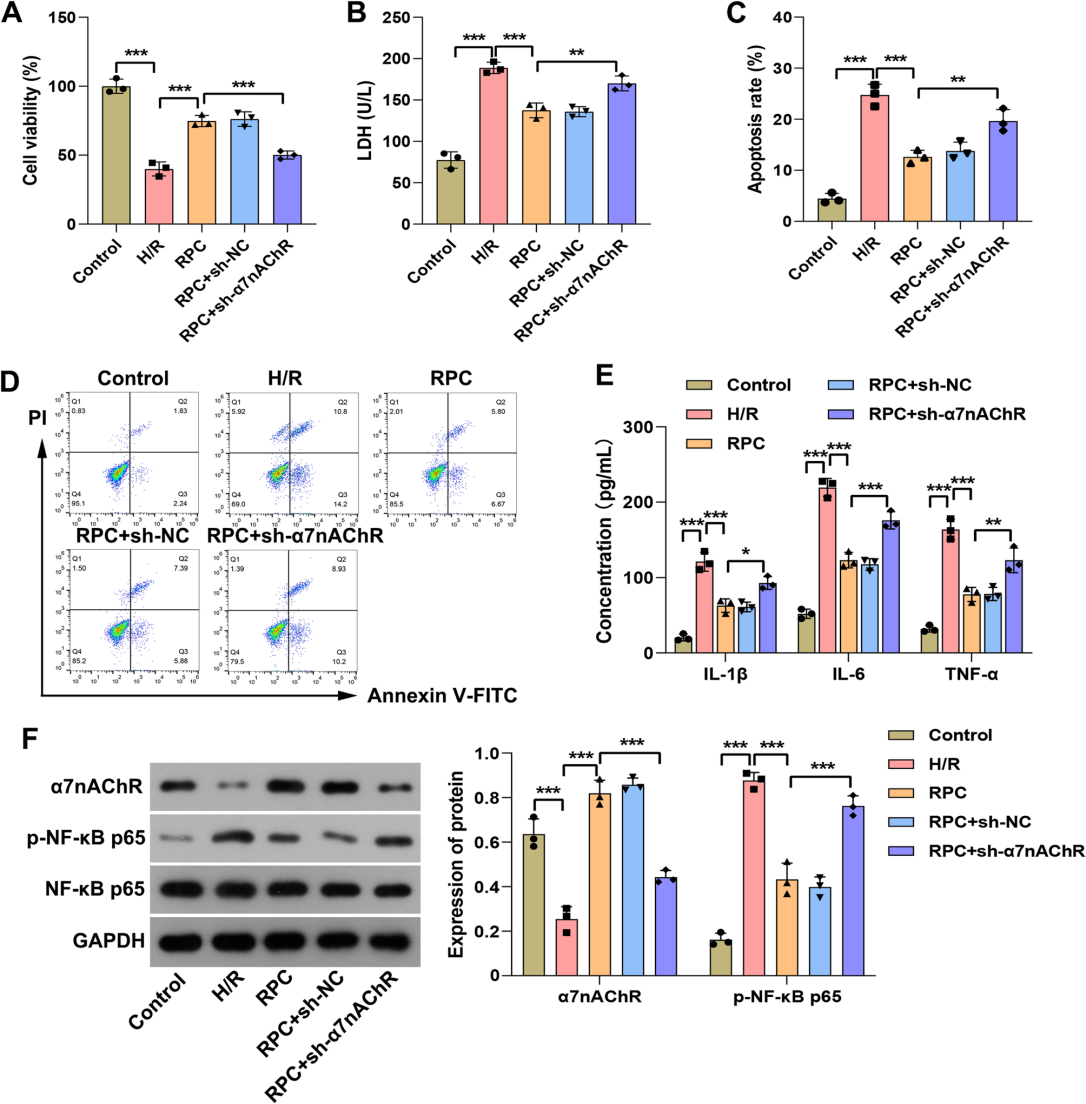
RPC alleviated H/R-exposed BRL-3 A injury by up-regulating α7nAChR. **A** The viability of BRL-3 A cells was assessed using the CCK-8 assay. **B** The activity of LDH in the culture medium of BRL-3 A cells were quantified using biochemical method. **C**-**D** The apoptosis rate of BRL-3 A cells was determined using flow cytometry analysis. **E** The concentrations of IL-1β, IL-6, and TNF-α in the culture medium of BRL-3 A cells were measured using ELISA. **F** The protein expression levels of α7nAChR, NF-κB p65, and p-NF-κB p65 in BRL-3 A cells was evaluated using Western blotting analysis. RPC was defined as 10 ng/mL remifentanil preconditioning. *n* = 3. * *p* < 0.05, ** *p* < 0.01, *** *p* < 0.001

To elucidate the specific protective mechanism of remifentanil against H/R-induced hepatocyte injury, loss-of-function experiments were conducted. Specifically, the expression of α7nAChR in BRL-3 A cells was silenced, followed by RPC and exposure to H/R. Initially, we evaluated the efficiency of three interfering lentiviral vectors and identified the most effective one. Among these, infection with sh-α7nAChR-3# resulted in the lowest expression levels of both α7nAChR mRNA and protein in BRL-3 A cells (Fig. 4C and D). Consequently, sh-α7nAChR-3# was selected for subsequent experiments to silence α7nAChR expression. The results demonstrated that the viability of BRL-3 A cells exposed to H/R was significantly reduced, while the levels of LDH in the cell culture medium were markedly elevated, along with an increased apoptosis rate. RPC enhanced the viability of BRL-3 A cells under H/R conditions (Fig. 5A), attenuated the activities of LDH in the cell culture medium (Fig. 5B), and decreased the apoptosis rate (Fig. 5C and D). Nevertheless, silencing of α7nAChR partially reversed the aforementioned protective effects of remifentanil. In addition, exposure of BRL-3 A cells to H/R resulted in a significant increase in the secretion of IL-1β, IL-6, and TNF-α. Furthermore, the phosphorylation level of NF-κB p65 was significantly upregulated, while the protein expression level of α7nAChR was decreased. Pretreatment with remifentanil markedly suppressed the secretion of these pro-inflammatory cytokines in H/R-exposed BRL-3 A cells (Fig. 5E), upregulated the expression of α7nAChR protein, and inhibited the phosphorylation of NF-κB p65 (Fig. 5F). Notably, knockdown of α7nAChR significantly diminished the inhibitory effects of remifentanil preconditioning on the release of pro-inflammatory cytokines and the phosphorylation of NF-κB p65. These results indicate that RPC mitigates H/R-induced hepatocyte injury, and the protective mechanism is associated with the up-regulation of α7nAChR to suppress the inflammatory response.

## Discussion

Hepatic I/R injury represents a complex pathophysiological process that leads to cellular damage and organ dysfunction, commonly observed as a complication following liver surgery. As the mechanistic understanding of hepatic I/R injury has become more in-depth and comprehensive, significant advancements have been achieved in its therapeutic approaches in recent years. Nevertheless, effective treatment strategies remain limited in clinical practice [2]. The present study simulated hepatic I/R injury at both in vivo and in vitro levels, demonstrating that remifentanil significantly alleviated liver tissue and cellular damage. Furthermore, through loss-of-function experiments, we identified that remifentanil inhibits the inflammatory response and apoptosis in hepatic I/R injury. Mechanistically, remifentanil upregulates the expression of α7nAChR in liver tissues and cells subjected to I/R injury, thereby suppressing the activation of the NF-κB signaling pathway and exerting anti-inflammatory effects. These findings provide a more comprehensive elucidation of the hepatoprotective mechanism of remifentanil, offering robust evidence for its potential application in clinical liver surgery.

In recent years, the hepatoprotective effects of remifentanil have garnered significant attention. Yang et al. demonstrated that RPC alleviates I/R liver injury both in vivo and in vitro by enhancing antioxidant and anti-inflammatory effects mediated by inducible nitric oxide synthase [22]. Furthermore, studies indicate that RPC mitigates I/R-induced liver injury through modulation of IL-18 signaling pathways [28]. Additionally, remifentanil serves as a negative regulator of Toll-like receptor 4 (TLR4)-mediated inflammatory responses, providing critical protection against hepatic I/R injury by inhibiting the TLR4 pathway to reduce inflammation and hepatocyte apoptosis [25]. These findings collectively underscore the substantial potential of RPC in attenuating hepatic I/R injury. Consequently, a thorough understanding of its mechanism of action is essential for its clinical application. ALT and AST are functional enzymes predominantly found in liver tissue [29]. Their elevated levels in the bloodstream typically indicate the presence of hepatic parenchymal injury [26, 30]. In the present study, rats subjected to hepatic I/R injury exhibited elevated serum levels of ALT and AST, increased production of proinflammatory cytokines, and enhanced necrotic and apoptotic cell death in liver tissue. Pretreatment with remifentanil significantly attenuated these I/R-induced liver injury characteristics. Therefore, consistent with the above findings, our experimental data also suggest that remifentanil plays an important hepatoprotective role during hepatic I/R injury. Notably, we observed that RPC upregulated the expression of α7nAChR in the injured liver tissue of I/R rats. Based on this observation, we propose that the hepatoprotective mechanism of RPC may involve the regulation of α7nAChR expression.

During the I/R process, the infiltration of inflammatory factors resulting from metabolic disorders and the activation of inflammatory signaling pathways serves as a critical factor contributing to liver tissue damage. In the ischemic phase, also referred to as the hypoxic phase, energy metabolism disturbances and the accumulation of reactive oxygen species (ROS) are observed. During the reperfusion period, characterized by oxygen restoration, a significant increase in ROS production occurs, accompanied by the migration of neutrophils and macrophages into liver tissue [31]. These immune cells release pro-inflammatory cytokines, including IL-1β, IL-6, and TNF-α [32]. ROS and inflammatory factors can directly induce hepatocyte damage, mediate apoptosis and necrosis of hepatocytes, and potentially trigger a systemic inflammatory response that affects distant organs [33]. NF-κB is a key transcription factor that responds to cellular stress and becomes activated under conditions of cellular hypoxia [34]. The NF-κB signaling pathway plays a pivotal role in mediating the inflammatory response associated with hepatic I/R injury. Activation of NF-κB triggers early inflammatory processes and amplifies the inflammatory cascade by modulating the expression of proinflammatory cytokines, chemokines, and cell adhesion molecules [32]. Inhibiting NF-κB activation can attenuate both the inflammatory response and apoptosis, thereby mitigating liver injury induced by I/R [35–37]. Consequently, targeting the NF-κB pathway represents a critical strategy for the treatment of hepatic I/R injury. The α7nAChR can directly or indirectly regulate NF-κB activation via multiple pathways. Specifically, α7nAChR promotes STAT3 phosphorylation, which inhibits the transcription of its target genes, including TNF-α, IL-6, and other pro-inflammatory cytokines, thereby indirectly reducing the stimulation signals that activate NF-κB [38, 39]. In the context of inflammation-related diseases, the activation of α7nAChR suppresses the activity of the IκB kinase complex, leading to the direct inhibition of NF-κB phosphorylation [40]. Additionally, as a ligand-gated calcium channel, α7nAChR induces calcium influx upon activation. The calcium signaling mediated by α7nAChR regulates NF-κB activity through calmodulin-dependent pathways, such as CaMKK/AMPK [41, 42]. It has been confirmed that the molecular mechanism by which α7nAChR exerts its anti-inflammatory effect through the inhibition of NF-κB activation plays a crucial role in protecting the liver against I/R injury [18]. In the present study, RPC was found to not only upregulate the expression of α7nAChR in I/R-damaged liver tissues and H/R-induced hepatocytes but also suppress the NF-κB phosphorylation. To further elucidate the underlying molecular mechanisms, loss-of-function experiments were conducted at both in vivo and in vitro levels. The findings demonstrated that the α7nAChR antagonist not only promoted NF-κB phosphorylation but also exacerbated liver inflammatory injury in rats subjected to I/R injury. Moreover, both the α7nAChR antagonist and α7nAChR knockdown partially reversed the protective effects of RPC on liver injury and H/R-induced hepatocyte injury in rats. These results collectively suggest that remifentanil alleviates liver injury during I/R by up-regulating α7nAChR to inhibit inflammatory response.

In conclusion, this study elucidates a novel molecular mechanism underlying the hepatoprotective effect of remifentanil and provides preliminary evidence that remifentanil alleviates inflammatory injury in liver tissue during I/R by modulating α7nAChR/NF-κB signaling. These findings enhance our understanding of the hepatoprotective mechanisms of remifentanil and offer new insights to facilitate its application as an anesthetic in liver surgery, such as hepatectomy and liver transplantation. There exists an interaction between the opioid and cholinergic systems. Evidence from studies indicates that acetylcholine can produce analgesic effects and contribute to the development of physical dependence through activation of the endogenous opioid system, a process mediated by α7nAChR [43, 44]. Consequently, remifentanil and α7nAChR activation exhibit synergistic effects in pain management [45]. Additionally, opioid receptor antagonists have been shown to non-competitively inhibit α7nAChR expression in rat hippocampal neurons [46]. However, in the context of liver ischemia-reperfusion injury, it remains unclear whether the interaction between opioids and the cholinergic system is consistent with the aforementioned findings. A key limitation of this study is the lack of mechanistic insight into how remifentanil activates α7nAChR. It is possible that remifentanil directly targets hepatic α7nAChR, or alternatively, exerts its effects indirectly through cellular stress pathways. These mechanisms warrant further investigation.

## Supplementary Information

Supplementary Material 1

Supplementary Material 2

## Data Availability

All data supporting the results of this study are available upon request from the corresponding author.

## References

[1] Konishi T, Schuster RM, Goetzman HS, Caldwell CC, Lentsch AB. Fibrotic liver has prompt recovery after ischemia-reperfusion injury. Am J Physiol Gastrointest Liver Physiol. 2020;318(3):G390–400. 31961717 10.1152/ajpgi.00137.2019 PMC7099490

[2] de Oliveira THC, Gonçalves GKN. Liver ischemia reperfusion injury: Mechanisms, cellular pathways, and therapeutic approaches. Int Immunopharmacol. 2025;150:114299. 39961215 10.1016/j.intimp.2025.114299

[3] Cannistrà M, Ruggiero M, Zullo A, Gallelli G, Serafini S, Maria M, et al. Hepatic ischemia reperfusion injury: A systematic review of literature and the role of current drugs and biomarkers. Int J Surg. 2016;33(Suppl 1):S57–70. 27255130 10.1016/j.ijsu.2016.05.050

[4] Zheng DF, Zha XJ, Jiang EL, Qiu Y, Yang W, Xiao WD. Trojan horse-like biohybrid nanozyme for ameliorating liver ischemia-reperfusion injury. Adv Healthc Mater. 2025;14(7):e2404458. 39828639 10.1002/adhm.202404458

[5] Noviello CM, Gharpure A, Mukhtasimova N, Cabuco R, Baxter L, Borek D, et al. Structure and gating mechanism of the α7 nicotinic acetylcholine receptor. Cell. 2021;184(8):2121–e21342113. 33735609 10.1016/j.cell.2021.02.049 PMC8135066

[6] Ren C, Tong YL, Li JC, Lu ZQ, Yao YM. The protective effect of alpha 7 nicotinic acetylcholine receptor activation on critical illness and its mechanism. Int J Biol Sci. 2017;13(1):46–56. 28123345 10.7150/ijbs.16404 PMC5264260

[7] Gallowitsch-Puerta M, Tracey KJ. Immunologic role of the cholinergic anti-inflammatory pathway and the nicotinic acetylcholine alpha 7 receptor. Ann N Y Acad Sci. 2005;1062:209–19. 16461803 10.1196/annals.1358.024

[8] Wu SJ, Shi ZW, Wang X, Ren FF, Xie ZY, Lei L, et al. Activation of the cholinergic anti-inflammatory pathway attenuated angiotension II-dependent hypertension and renal injury. Front Pharmacol. 2021;12:593682. 33815099 10.3389/fphar.2021.593682 PMC8010129

[9] Chen Y, Zhang Y, Wang J, Li S, Wang Y, Zhang Z, et al. Anti-neuroinflammation effects of transcutaneous auricular vagus nerve stimulation against depression- like behaviors via hypothalamic α7nAchR/JAK2/STAT3/NF-κB pathway in rats exposed to chronic unpredictable mild stress. CNS Neurosci Ther. 2023;29(9):2634–44. 37032645 10.1111/cns.14207 PMC10401149

[10] Sun Y, Jia D, Xue M, Huang Z, Huang C. Trifluoro-icaritin alleviates chronic inflammatory pain through α7nAChR-mediated suppression of HMGB1/NF-κB signaling in the spinal cord of rats. Brain Res Bull. 2022;183:13–26. 35202753 10.1016/j.brainresbull.2022.02.014

[11] Han Z, Shen F, He Y, Degos V, Camus M, Maze M, et al. Activation of α-7 nicotinic acetylcholine receptor reduces ischemic stroke injury through reduction of pro-inflammatory macrophages and oxidative stress. PLoS ONE. 2014;9(8):e105711. 25157794 10.1371/journal.pone.0105711 PMC4144901

[12] Han Z, Li L, Wang L, Degos V, Maze M, Su H. Alpha-7 nicotinic acetylcholine receptor agonist treatment reduces neuroinflammation, oxidative stress, and brain injury in mice with ischemic stroke and bone fracture. J Neurochem. 2014;131(4):498–508. 25040630 10.1111/jnc.12817 PMC4221541

[13] Zhang Y, Ma R, Wang W, Deng Q, Cao C, Yu C, et al. Activation of α7nAChR by PNU282987 improves cognitive impairment through inhibiting oxidative stress and neuroinflammation in D-galactose induced aging via regulating α7nAChR/ Nrf2/HO-1 signaling pathway. Exp Gerontol. 2023;175:112139. 36898594 10.1016/j.exger.2023.112139

[14] Khalaf HM, Ahmed SM, Welson NN, Abdelzaher WY. Rivastigmine ameliorates indomethacin experimentally induced gastric mucosal injury via activating α7nAChR with inhibiting oxidative stress and apoptosis. J Biochem Mol Toxicol. 2022;36(10):e23147. 35702939 10.1002/jbt.23147

[15] Wang H, Cai D, Chen Z, Wang Y. GTS-21 promotes α7 nAChR to alleviate intestinal ischemia-reperfusion-induced apoptosis and inflammation of enterocytes. Med Sci Monit. 2020;26:e921618. 32417847 10.12659/MSM.921618 PMC7251968

[16] Xiong J, Yuan YJ, Xue FS, Wang Q, Cheng Y, Li RP, et al. Postconditioning with α7nAChR agonist attenuates systemic inflammatory response to myocardial ischemia–reperfusion injury in rats. Inflammation. 2012;35(4):1357–64. 22391744 10.1007/s10753-012-9449-2

[17] Ding L, Ye H, Gu LD, Du AQ, Yuan XL. Echinacoside alleviates cognitive impairment in cerebral ischemia rats through α 7nAChR-induced autophagy. Chin J Integr Med. 2022;28(9):809–16. 35799084 10.1007/s11655-022-2893-4

[18] Li F, Chen Z, Pan Q, Fu S, Lin F, Ren H, et al. The protective effect of PNU-282987, a selective α7 nicotinic acetylcholine receptor agonist, on the hepatic ischemia-reperfusion injury is associated with the Inhibition of high- mobility group box 1 protein expression and nuclear factor κB activation in mice. Shock. 2013;39(2):197–203. 23324890 10.1097/SHK.0b013e31827aa1f6

[19] Zhou B, Liu J, Jin L, Huang X. Remimazolam alleviates hepatic ischemia- reperfusion injury by activating FOXO1/3 signaling: remimazolam alleviates hepatic ischemia reperfusion injury. BMC Gastroenterol. 2025;25(1):283. 40263992 10.1186/s12876-025-03820-3 PMC12016092

[20] Bedirli N, Ofluoglu E, Kerem M, Utebey G, Alper M, Yilmazer D, et al. Hepatic energy metabolism and the differential protective effects of Sevoflurane and isoflurane anesthesia in a rat hepatic ischemia-reperfusion injury model. Anesth Analg. 2008;106(3):830–7. 18292427 10.1213/ane.0b013e3181616fc9

[21] Shan H, Wang Z, Chen Y, Ma TF, Zhang J, Zhang J, et al. Etomidate inhibits hepatic ischemia-reperfusion injury depending on the activation of Nrf2-HO-1 signaling pathway. DNA Cell Biol. 2025;44(1):13–24. 39470379 10.1089/dna.2024.0125

[22] Yang LQ, Tao KM, Liu YT, Cheung CW, Irwin MG, Wong GT, et al. Remifentanil preconditioning reduces hepatic ischemia-reperfusion injury in rats via inducible nitric oxide synthase expression. Anesthesiology. 2011;114(5):1036–47. 21383616 10.1097/ALN.0b013e3182104956

[23] Chen CR, Bi HL, Li X, Li ZM. Remifentanil protects neurological function of rats with cerebral ischemia-reperfusion injury via NR2B/CaMKIIα signaling pathway. J Biol Regul Homeost Agents. 2020;34(5):1647–56. 33103411 10.23812/20-169-A

[24] Li J, Wang S, Huang S, Shao W, Zhang J. Remifentanil anesthesia on the expression of apoptosis-related proteins Bcl-2 and Bax in rat myocardial cells with ischemia-reperfusion injury. Cell Mol Biol (Noisy-le-grand). 2022;67(5):96–103. 35818266 10.14715/cmb/2021.67.5.13

[25] Yang Y, Chen C, Cui C, Jiao Y, Li P, Zhu L, et al. Indispensable role of β-arrestin2 in the protection of remifentanil preconditioning against hepatic ischemic reperfusion injury. Sci Rep. 2019;9(1):2087. 30765766 10.1038/s41598-018-38456-9 PMC6376065

[26] Park J, Kang JW, Lee SM. Activation of the cholinergic anti-inflammatory pathway by nicotine attenuates hepatic ischemia/reperfusion injury via Heme oxygenase-1 induction. Eur J Pharmacol. 2013;707(1–3):61–70. 23535606 10.1016/j.ejphar.2013.03.026

[27] Qian B, Yin B, Yu H, Wang C, Lu S, Ke S, et al. Axin formation inhibitor 1 aggravates hepatic ischemia–reperfusion injury by promoting the ubiquitination and degradation of PPARβ. Nat Commun. 2025;16(1):1776. 39971912 10.1038/s41467-025-56967-8 PMC11840116

[28] Liu X, Pan Z, Su D, Yang Z, Zheng B, Wang X, et al. Remifentanil ameliorates liver ischemia-reperfusion injury through Inhibition of interleukin-18 signaling. Transplantation. 2015;99(10):2109–17. 25919765 10.1097/TP.0000000000000737

[29] van Beek JH, de Moor MH, de Geus EJ, Lubke GH, Vink JM, Willemsen G, et al. The genetic architecture of liver enzyme levels: GGT, ALT and AST. Behav Genet. 2013;43(4):329–39. 23580007 10.1007/s10519-013-9593-y PMC3918238

[30] Chinnappan R, Mir TA, Alsalameh S, Makhzoum T, Adeeb S, Al-Kattan K, et al. Aptasensors are conjectured as promising ALT and AST diagnostic tools for the early diagnosis of acute liver injury. Life. 2023. 10.3390/life13061273. 37374056 10.3390/life13061273 PMC10305476

[31] Hirao H, Nakamura K, Kupiec-Weglinski JW. Liver ischaemia-reperfusion injury: a new Understanding of the role of innate immunity. Nat Rev Gastroenterol Hepatol. 2022;19(4):239–56. 34837066 10.1038/s41575-021-00549-8

[32] Dossi CG, Vargas RG, Valenzuela R, Videla LA. Beneficial effects of natural compounds on experimental liver ischemia-reperfusion injury. Food Funct. 2021;12(9):3787–98. 33977997 10.1039/d1fo00289a

[33] Kadkhodaee M, Mikaeili S, Zahmatkesh M, Golab F, Seifi B, Arab HA, et al. Alteration of renal functional, oxidative stress and inflammatory indices following hepatic ischemia-reperfusion. Gen Physiol Biophys. 2012;31(2):195–202. 22781823 10.4149/gpb_2012_024

[34] D’Ignazio L, Rocha S. Hypoxia induced NF-κB. Cells. 2016;5(1):10. 10.3390/cells5010010 PMC4810095 27005664

[35] Mou T, Luo Y, Huang Z, Zheng D, Pu X, Shen A, et al. Inhibition of microRNA-128-3p alleviates liver ischaemia-reperfusion injury in mice through repressing the Rnd3/NF-κB axis. Innate Immun. 2020;26(6):528–36. 32486927 10.1177/1753425920928449 PMC7491242

[36] Ding J, WenjuanYang, Jiang Y, Ji J, Zhang J, Wu L, et al. Cordycepin protects against hepatic ischemia/reperfusion injury via inhibiting MAPK/NF-κB pathway. Mediators Inflamm. 2022;2022:5676256. 36518880 10.1155/2022/5676256 PMC9744625

[37] Ibrahim MA, Abdelzaher WY, Ibrahim YF, Ahmed AF, Welson NN, Al-Rashed S, et al. Diacerein protects rats with liver ischemia/reperfusion damage: Down- regulation of TLR4/NFκ-B signaling pathway. Biomed Pharmacother. 2021;134:111063. 33348310 10.1016/j.biopha.2020.111063

[38] Lei W, Zhao C, Sun J, Jin Y, Duan Z. Activation of α7nAChR preserves intestinal barrier integrity by enhancing the HO-1/STAT3 signaling to inhibit NF-κB activation in mice. Biomed Pharmacother. 2022;149:112733. 36068769 10.1016/j.biopha.2022.112733

[39] Guo H, Jin D, Chen X. Lipocalin 2 is a regulator of macrophage polarization and NF-κB/STAT3 pathway activation. Mol Endocrinol. 2014;28(10):1616–28. 25127375 10.1210/me.2014-1092 PMC4179633

[40] Yuan F, Jiang L, Li Q, Sokulsky L, Wanyan Y, Wang L, et al. A selective α7 nicotinic acetylcholine receptor agonist, PNU-282987, attenuates ILC2s activation and alternaria-induced airway inflammation. Front Immunol. 2020;11:598165. 33597946 10.3389/fimmu.2020.598165 PMC7883686

[41] Zhao J, Yu L, Xue X, Xu Y, Huang T, Xu D, et al. Diminished α7 nicotinic acetylcholine receptor (α7nAChR) rescues amyloid-β induced atrial remodeling by oxi-CaMKII/MAPK/AP-1 axis-mediated mitochondrial oxidative stress. Redox Biol. 2023;59:102594. 36603528 10.1016/j.redox.2022.102594 PMC9813735

[42] Hattori K, Takahashi N, Terabe K, Ohashi Y, Kishimoto K, Yokota Y, et al. Activation of transient receptor potential vanilloid 4 protects articular cartilage against inflammatory responses via CaMKK/AMPK/NF-κB signaling pathway. Sci Rep. 2021;11(1):15508. 34330980 10.1038/s41598-021-94938-3 PMC8324869

[43] Kishioka S, Kiguchi N, Kobayashi Y, Saika F. Nicotine effects and the endogenous opioid system. J Pharmacol Sci. 2014;125(2):117–24. 24882143 10.1254/jphs.14r03cp

[44] Ueno K, Kiguchi N, Kobayashi Y, Saika F, Wakida N, Yamamoto C, et al. Possible involvement of endogenous opioid system located downstream of α7 nicotinic acetylcholine receptor in mice with physical dependence on nicotine. J Pharmacol Sci. 2014;124(1):47–53. 24366190 10.1254/jphs.13172fp

[45] Gu W, Zhang W, Lei Y, Cui Y, Chu S, Gu X, et al. Activation of spinal alpha-7 nicotinic acetylcholine receptor shortens the duration of remifentanil-induced postoperative hyperalgesia by upregulating KCC2 in the spinal dorsal Horn in rats. Mol Pain. 2017;13:1744806917704769. 28425312 10.1177/1744806917704769 PMC6997724

[46] Almeida LE, Pereira EF, Alkondon M, Fawcett WP, Randall WR, Albuquerque EX. The opioid antagonist Naltrexone inhibits activity and alters expression of alpha7 and alpha4beta2 nicotinic receptors in hippocampal neurons: implications for smoking cessation programs. Neuropharmacology. 2000;39(13):2740–55. 11044744 10.1016/s0028-3908(00)00157-x

