# Supplementary material for: Involvement of α7nAChR in the hepatic-protective effect of remifentanil preconditioning in ischemia/reperfusion rats
Source: Hereditas. 2025 Dec 29;162:239. doi: 10.1186/s41065-025-00601-6 (PMC12751796; doi:10.1186/s41065-025-00601-6)
Supplement: Supplementary file 1 — Supplementary Material 1 [file 41065_2025_601_MOESM1_ESM.pdf]

Figure 2B

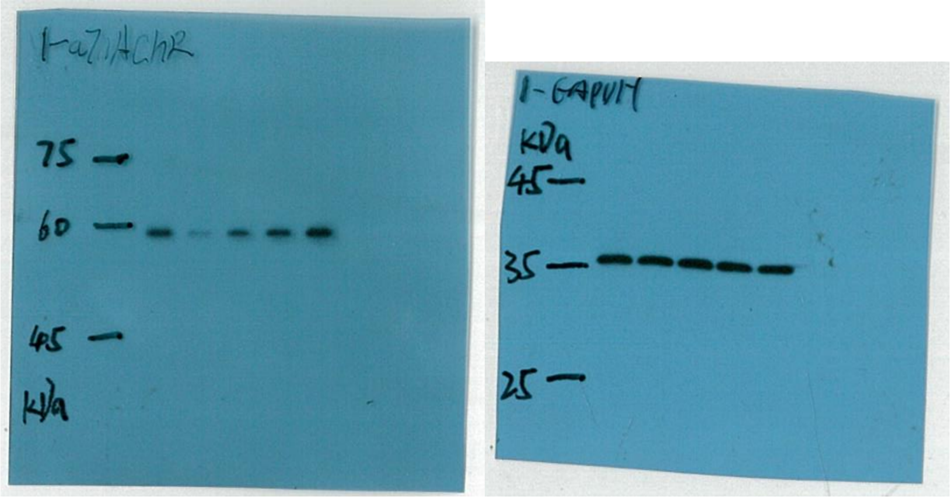

Lane 1, Sham group; lane 2, I/R group; lane 3, RPC1 group; lane 4, RPC2 group; lane 5, RPC5 group.

Figure 3A

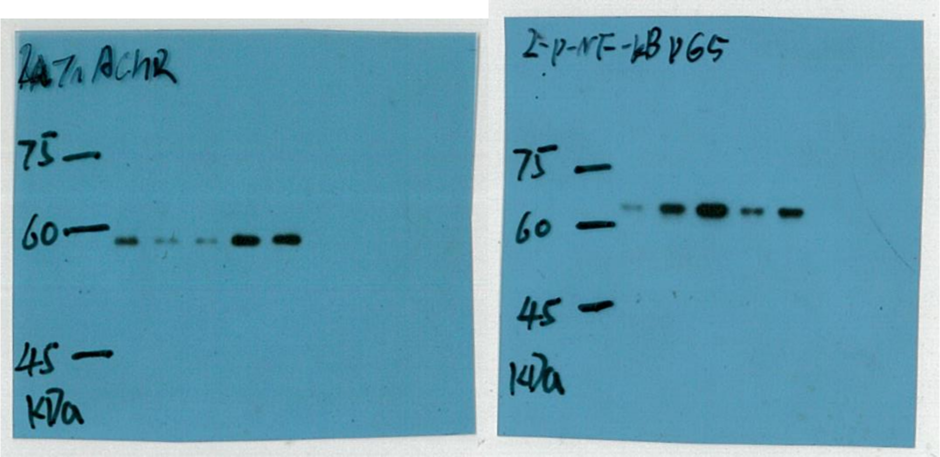

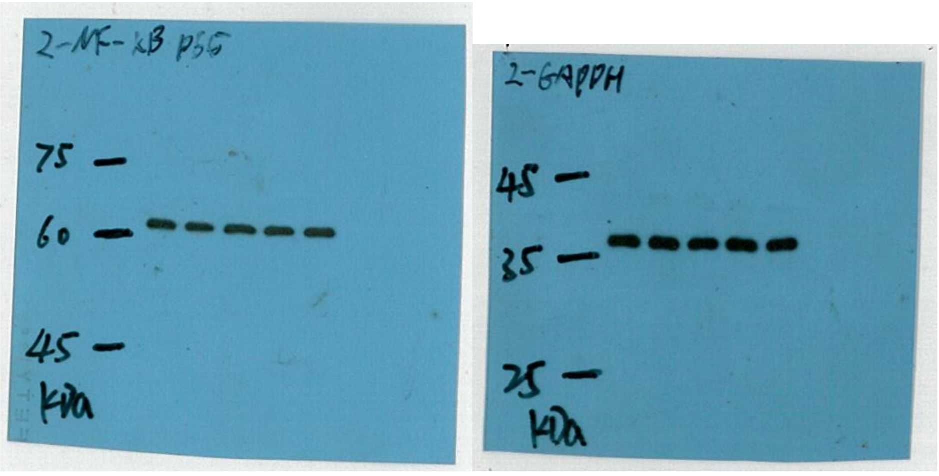

Lane 1, Sham group; lane 2, I/R group; lane 3, MLA group; lane 4, RPC group; lane 5, RPC+MLA group.

Figure 4B

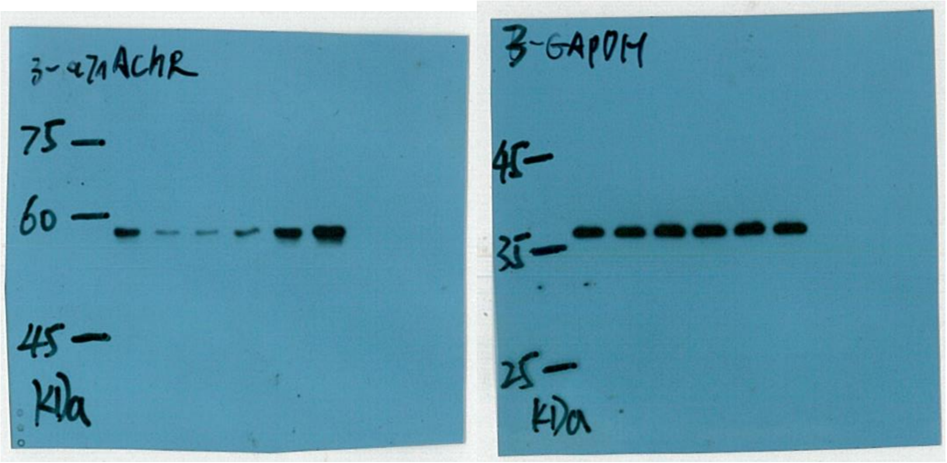

Lane 1, control group; lane 2, H/R group; lane 3, H/R+0.1 ng/mL RPC group; lane 4, H/R+1 ng/mL RPC group; lane 5, H/R+10 ng/mL RPC group.

Figure 4C

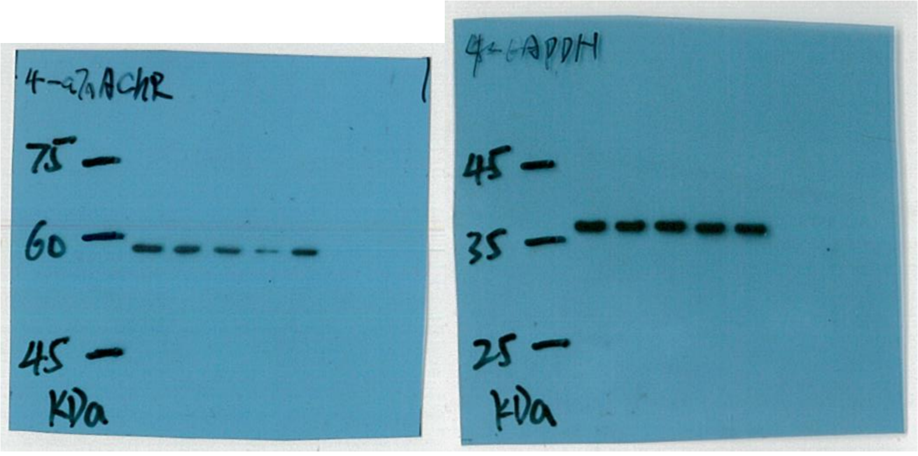

Lane 1, blank group; lane 2, sh-α7nAChR-1# group; lane 3, sh-α7nAChR-2# group; lane 4, sh-α7nAChR-3# group; lane 5, sh-NC group.

Figure 5E

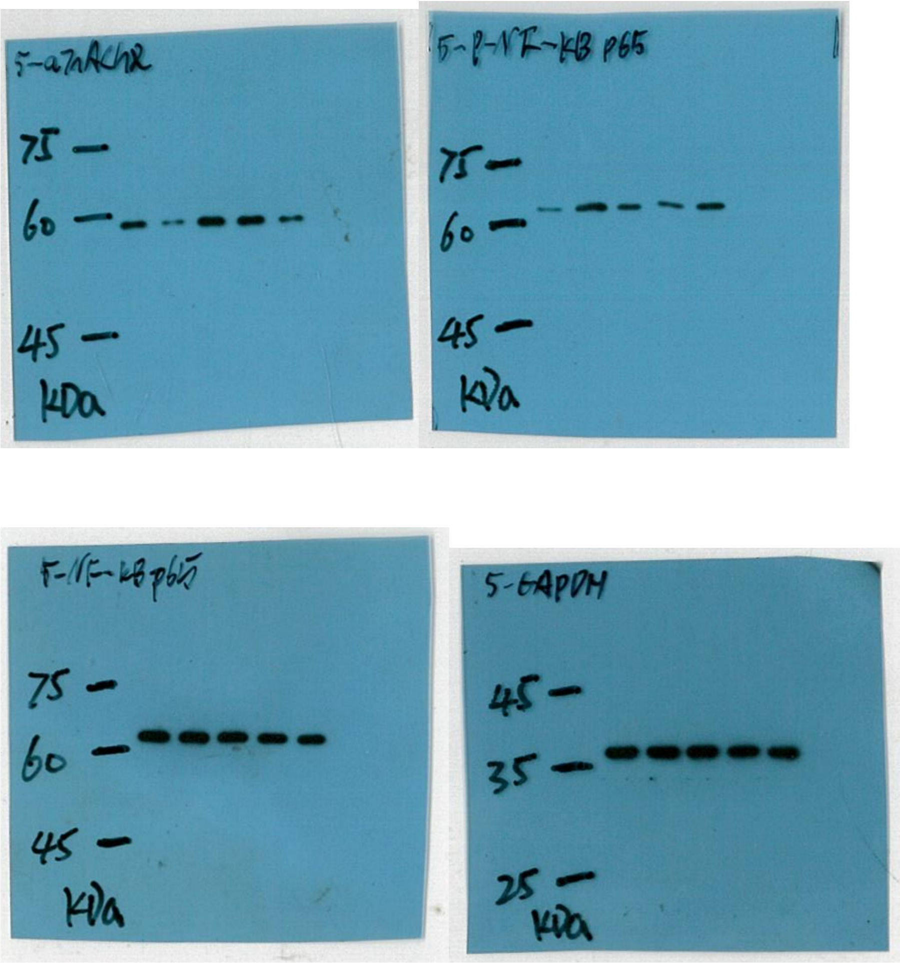

Lane 1, control group; lane 2, H/R group; lane 3, RPC group; lane 4, RPC+sh-NC group; lane 5, RPC+ sh-α7nAChR group.
