# Supplementary material for: Involvement of α7nAChR in the hepatic-protective effect of remifentanil preconditioning in ischemia/reperfusion rats
Source: Hereditas. 2025 Dec 29;162:239. doi: 10.1186/s41065-025-00601-6 (PMC12751796; doi:10.1186/s41065-025-00601-6)
Supplement: Supplementary file 2 — Supplementary Material 2 [file 41065_2025_601_MOESM2_ESM.pdf]

伦理批件号：HG-KY-2024-035

伦 理 证 明

本论文:《α7nAChR 在瑞芬太尼预处理缺血/再灌注大鼠肝脏保护作用中的参与》，动物实验研究部分，将自觉遵守实验动物福利伦理原则，同意接受委员会或实验室管理者的监督与检查，如违反承诺和规定，自愿接受处罚。

研究过程中:

1、在不影响实验结果科学性、可比性的前提下，用低进化水平的动物替代高级动物或者用组织细胞替代整体动物；

2、尽量减少不必要的动物使用数量；

3、合理设计实验方案以保护实验动物。

经黄冈市中心医院伦理委员会全体委员讨论，一致通过，该论文动物研究部分符合医学伦理学要求。

特此证明。

伦理委员会主任委员（签名）：

黄冈市中心医院伦理委员会（盖章）

日期：2023 年 10 月 20 日
